# Lessons Learned From Interdisciplinary Efforts to Combat COVID-19 Misinformation: Development of Agile Integrative Methods From Behavioral Science, Data Science, and Implementation Science

**DOI:** 10.2196/40156

**Published:** 2023-02-03

**Authors:** Sahiti Myneni, Paula Cuccaro, Sarah Montgomery, Vivek Pakanati, Jinni Tang, Tavleen Singh, Olivia Dominguez, Trevor Cohen, Belinda Reininger, Lara S Savas, Maria E Fernandez

**Affiliations:** 1 School of Biomedical Informatics The University of Texas Health Science Center Houston, TX United States; 2 Department of Health Promotion & Behavioral Sciences School of Public Health The University of Texas Health Science Center Houston, TX United States; 3 Center for Health Promotion and Prevention Research School of Public Health The University of Texas Health Science Center Houston, TX United States; 4 The University of Texas Health Science Center Tyler, TX United States; 5 Department of Biomedical Informatics and Medical Education The University of Washington Seattle, WA United States; 6 School of Public Health Brownsville Regional Campus The University of Texas Health Science Center Brownsville, TX United States

**Keywords:** COVID-19, misinformation, social media, health belief model, deep learning, community engagement

## Abstract

**Background:**

Despite increasing awareness about and advances in addressing social media misinformation, the free flow of false COVID-19 information has continued, affecting individuals’ preventive behaviors, including masking, testing, and vaccine uptake.

**Objective:**

In this paper, we describe our multidisciplinary efforts with a specific focus on methods to (1) gather community needs, (2) develop interventions, and (3) conduct large-scale agile and rapid community assessments to examine and combat COVID-19 misinformation.

**Methods:**

We used the Intervention Mapping framework to perform community needs assessment and develop theory-informed interventions. To supplement these rapid and responsive efforts through large-scale online social listening, we developed a novel methodological framework, comprising qualitative inquiry, computational methods, and quantitative network models to analyze publicly available social media data sets to model content-specific misinformation dynamics and guide content tailoring efforts. As part of community needs assessment, we conducted 11 semistructured interviews, 4 listening sessions, and 3 focus groups with community scientists. Further, we used our data repository with 416,927 COVID-19 social media posts to gather information diffusion patterns through digital channels.

**Results:**

Our results from community needs assessment revealed the complex intertwining of personal, cultural, and social influences of misinformation on individual behaviors and engagement. Our social media interventions resulted in limited community engagement and indicated the need for consumer advocacy and influencer recruitment. The linking of theoretical constructs underlying health behaviors to COVID-19–related social media interactions through semantic and syntactic features using our computational models has revealed frequent interaction typologies in factual and misleading COVID-19 posts and indicated significant differences in network metrics such as degree. The performance of our deep learning classifiers was reasonable, with an F-measure of 0.80 for speech acts and 0.81 for behavior constructs.

**Conclusions:**

Our study highlights the strengths of community-based field studies and emphasizes the utility of large-scale social media data sets in enabling rapid intervention tailoring to adapt grassroots community interventions to thwart misinformation seeding and spread among minority communities. Implications for consumer advocacy, data governance, and industry incentives are discussed for the sustainable role of social media solutions in public health.

## Introduction

Exposure to COVID-19 health misinformation has emerged as a global risk factor for human health and wellness [1]. Expanding mobile connectivity and access to digital media allows for the dissemination of both evidence-based and unvetted resources online in this increasingly connected information environment. COVID-19 is the first global pandemic during this social media era, revealing several key shifts in health information consumption by the general public that challenge traditional knowledge and remediation pathways to combat health misinformation [2-5]. Studies show that (1) health consumers are no longer passive readers, but active contributors of misinformation seeding and spread; (2) such contributions can be unintentional and stem from anywhere in the world, affecting the public’s perceptions, behaviors, and potential COVID-19–related risks; (3) contamination with other information verticals, including politics, global monetization of media corporations, and inconsistent public health responses around the globe, can multiply mistrust in scientific institutions; and (4) increasing reliance on artificial intelligence and automated content recommendation algorithms confine people to opinion bubbles and echo chambers, with little human moderation, ultimately resulting in polarized social circles that make misinformation easy to proliferate [6-9].

Another striking observation is that COVID-19 misinformation traveled much faster than truth [10-14]. According to a recent report, 20% of COVID-19 misinformation comes from high-profile accounts (celebrities, politicians, and talk radio personalities) and 80% comes from the general public, with the former capturing much higher engagement rates (69% compared to 31%) [15]. Containing misinformation spread is further complicated by rapidly changing public health recommendations that follow emerging COVID-19 research. For instance, mask wearing recommendations have changed throughout the pandemic, resulting in confusion and misinformation associated with mask-wearing behavior [16] as well as mistrust among the population about the validity of recommendations [17].

Research has shown that exposure to COVID-19 misinformation is associated with age, education, and income levels of an individual [18]. Previous studies have also identified main themes related to the spread of COVID-19 misinformation in social media and how it fluctuated with time, for example, there were false stories about the source of the virus in the beginning of the pandemic, followed by false information spread about home remedies, etc [19]. Misinformation about COVID-19 can lead to increased risk of exposure and susceptibility to the virus (eg, promoting vaccine hesitancy), thus affecting the global course of the pandemic. To this end, emerging research suggests that misinformation modeling and management should be considered a critical component of public health campaigns and interventions [20] because of the various dynamics involving information exposure, human behavior, and disease spread. Current tools that automate misinformation detection are prone to algorithmic bias and offer little or no context for individuals to engage in self-reflection and recalibration of their health beliefs, attitudes, and latent heuristics, bringing into question the credibility, equity, and cultural appropriateness of such tools [21-23].

In this paper, our aim is to describe our interdisciplinary efforts to combat COVID-19 misinformation, which include needs assessment, misinformation modeling, and intervention development and dissemination. For the purpose of this work, we used mixed methods community needs assessment and leveraged recent advances in social computing and data science. These methods enable us to conduct large-scale online social listening and gain granular understanding of community needs. Further, these methods allow dissemination of evidence-based information in online settings and at-risk communities in the field to promote COVID-19 testing and vaccination for general and minority populations. In subsequent sections of this paper, we describe how the methods and results of our community needs assessment, integration of behavioral theory, social computing techniques, and social network analysis contributed to COVID-19–related knowledge discovery and interventions. This article aims to help public health researchers, social marketing teams, implementation scientists, disease prevention and health disparity experts, informaticians, and social media technologists expand their understanding of qualitative methods and data science tools, and highlight the missed opportunities in appropriately leveraging these resources for public health and wellness during the COVID-19 pandemic.

## Methods

### Intervention Mapping

We used the Intervention Mapping (IM) framework [24] that offers a systematic approach to intervention development and adaptation. IM is designed to develop multilevel interventions, such as the one described, in that it considers not only the behavior (COVID-19 testing and vaccination) but also the interpersonal environment (social marketing to promote COVID-19–related protective behaviors). The IM process comprises the following 6 steps: (1) conducting a needs and assets assessment to create a logical model of the problem for stating intervention goals; (2) flipping the logical model of the problem into the logic model of change by identifying the behavioral and environmental outcomes for the intervention; (3) designing the intervention with theory- and evidence-based change methods; (4) developing the intervention products with a process of pretesting, refining, and producing intervention materials; (5) implementing the intervention plan by identifying potential program users and program performance objectives; and (6) developing indicators and measures for intervention evaluation. In this paper, we focus on IM steps 1, 2, and 4 given the methodological focus, and outlining our activities for rigorous implementation, process evaluation, and effects evaluation is outside the scope of this effort.

**Figure 1:**
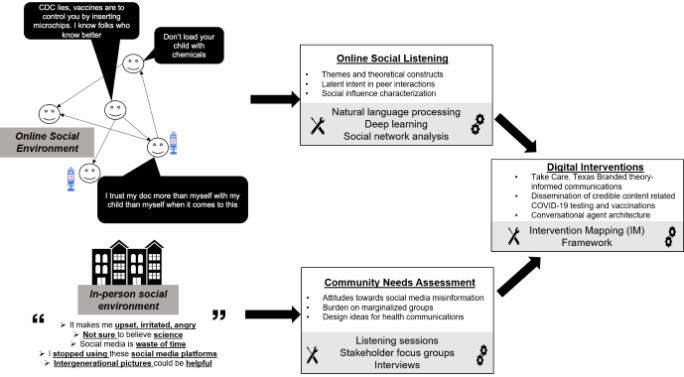
Overall research methodology. CDC: Centers for Disease Control and Prevention.

Figure 1 illustrates the multilevel nature of our methodology to identify and combat COVID-19 misinformation as described in the sections below.

### Community Needs Assessment

We collected information from multiple sources as part of our community needs assessment. We conducted virtual interviews with participants (n=11) to understand attitudes, beliefs, and knowledge about COVID-19 testing and vaccines in the context of usability evaluation of digital learning environments (eg, chatbots and existing social media) to address misinformation. We recruited participants using direct contact and social media advertising, and they were provided with a description of the study and an informed consent form. Once the consent form was signed, a team of 2 researchers conducted interviews. On average, each interview lasted 32 minutes and was digitally recorded. Once transcribed, all interviews were analyzed with the methods of directed content analysis [25] using Dedoose software (SocioCultural Research Consultants). Each participant who participated in the interview received US $25 compensation. From April to July 2021, local nonprofit agencies trained by the civic engagement group hosted and facilitated 7 listening sessions with nearly 70 community members in areas identified as heavily impacted by COVID-19. The sessions prioritized the experiences of those living in predominantly Black and Latinx neighborhoods, those in refugee and immigrant communities, those in low-income households, young adults, and those whose primary language is Spanish. Participants were asked about motivators, hesitations, structural barriers, rumors, and misinformation pertaining to COVID-19 vaccines. Each participant who attended a listening session received US $50 compensation for participating. The civic organization shared their analysis to inform our intervention development. We subsequently gathered input from stakeholders in 3 meetings with community scientists (around 12-15 participants in each session) from May 2021 to February 2022. The community scientist program is part of the National Institutes of Health–funded Center for Clinical and Translational Sciences to provide feedback from community members trained to understand scientific reasoning about aspects of the research process. Our sessions focused on the applicability and cultural appropriateness of our existing COVID-19 education and intervention materials. Our focus on minority populations was limited to qualitative inquiry, that is, listening sessions and interviews. Our social computing methods described in the next section capture the views of the general population. It is important to note that minority participants from our community needs assessment mentioned the use of specific social media platforms (eg, Facebook, Twitter, and YouTube) where they routinely were exposed to COVID-19 misinformation. Based on this insight, we conducted deeper secondary analysis of online social discourse to examine and portray the sociobehavioral mechanisms underlying misinformation spread and social resistance pathways.

#### Ethical Considerations

Our virtual interviews were deemed exempt by the institutional review board at the University of Texas Health Science Center at Houston (HSC-SBMI-18-1003). Neither community listening sessions nor Community Scientist sessions underwent review from an institutional review board. The information we present here is with the consent of the organizations involved.

### Online Social Listening

We used 2 distinct online social discourse data sets for this analysis. Using a public COVID-19 tweet-ID repository [26], we retrieved tweets published from January 2020 to January 2021. Tweets were hydrated using Twitter’s application programming interface (API) and the Twarc package [27], resulting in a total of 416,927 English-language tweets. We only used the original tweets (ie, excluding retweets and quotes) in our analysis. From these, a subset of 1400 tweets was randomly selected for further qualitative analysis as described below. To calculate the interrater reliability, a subset of 100 tweets was initially coded by 2 researchers, and any disagreements were mutually resolved via discussions between the 2 researchers to determine the appropriate label before proceeding with additional coding.

In addition, we employed the COVID-19 Twitter misinformation data set called CMU-MisCov19 [27] that was created to characterize COVID-19–related information in online social media to ensure robustness in our modeling efforts. This data set consisted of a total of 4573 Twitter IDs annotated for 17 categories, including tweets calling out or correcting misinformation, false public health response, false fact or prevention, true public health response, true prevention, etc [27]. We hydrated the tweets using Twitter’s API and the Twarc package [26], resulting in a final data set of 3702 tweets. Of these, a total of 1204 tweets exhibited misinformation resistance, in which the users were specifically calling out or correcting COVID-19 misinformation (ie, the stance taking corrective tweets).

#### Ethical Considerations

Our social media analysis was reviewed and deemed exempt by our institutional review board at the University of Texas Health Science Center at Houston (HSC-SBMI-15-0697).

#### Content and Intent Characterization

We coded the tweets using a list of constructs included in health behavior theories, including the Health Belief Model (HBM), Social Cognitive Theory, and Theory of Planned Behavior [28-31]. Examples of those constructs include perceived severity, cues to action, social norms, and self-efficacy [32]. For illustration purposes, in this paper, we present our analysis of HBM-related constructs using our high-throughput social computing methods (ie, methods that can be scaled to large volume data sets obtained from social media platforms). To understand the health beliefs associated with COVID-19 spread and misinformation in online social media platforms, we used a subset of 1400 tweets (7%) selected at random from a filtered set of 20,000 high-impact tweets (depending on their dissemination levels such as likes, retweets, etc) obtained from a total of 416,927 tweets. We analyzed every tweet within it for the manifestation of constructs outlined in the HBM. To understand how online users express their latent intent toward COVID-19 misinformation, we used a modified version of Searle’s speech act theory [33] and manually coded tweets for various categories of speech acts (eg, declaratives, stance, and assertion). Identification of speech acts in social media content provides a deeper insight into the interactions among individuals derived from attitudes toward topics and actions conveyed through language [33]. The detailed definitions of speech acts and their examples can be found in a previous report [34]. Tweets not falling into any of the categories were labeled as not applicable (NA). The interrater reliability was 0.81 (Cohen kappa) for HBM labels and 0.84 (Krippendorff alpha) for speech act labels.

#### Social Influence Characterization

The social influence of the tweets was captured via different dissemination levels based on their audience size and popularity. A tweet’s audience size was derived from the follower count, and its popularity was reflected by the number of retweets and likes/favorites, which propagate the tweet to other users [35]. The sum of these quantities indicated the total number of user interactions with each tweet. For the CMU-MisCov19 data set, dissemination levels were assigned based on tweet-level metrics capturing users’ interactions with the tweets (in this case, retweets and favorites), and tweets were classified as follows: “high” dissemination level (>11 interactions), “low” dissemination level (1-11 interactions), and “no” dissemination level (0 interactions). There were 527 tweets with high dissemination, 1593 with low dissemination, and 1582 with no dissemination in the data set.

#### Deep Learning Classification

To capture the population-level insights as our society navigated the course of the pandemic through the use of digital media, we used deep learning methods to scale the extraction of health beliefs and speech acts embedded within Twitter user interactions. Such methods have already been applied by researchers to capture the health beliefs associated with health-related conditions [36]. In this study, we evaluated the performance of the following models for classification of the HBM constructs and speech acts embedded within the data sets: (1) BERTweet [37], (2) BERTweet-Covid19 [37], and (3) ensemble of the 2 models (BERTweet+BERTweet-Covid19). These models are the result of unsupervised pretraining on tweets, providing a model with general linguistic information that can then be used by a classification module appended to it. Using the manually coded data set (n=1400 tweets), we first performed text preprocessing in order to convert the text to lowercase and also remove any hyperlinks from the textual data. We then split the entire data set into 90%, 5%, and 5% sets for training, validation, and testing, respectively. We used a learning rate of 1×10^−5^. We also computed class weights for the loss function to assign a higher weight to the loss encountered by tweets associated with minor classes (ie, the labels that had a lower prevalence as compared to the labels that had a higher prevalence in the given data set). The model was trained for 20 epochs. We converted the probabilities into actual classes based on the threshold value calculated using the validation set. We used recall, precision, and F1-score to evaluate the classifier’s predictions on the held-out test data set. Based on the prevalence of various categories of HBM constructs in the manually coded data set, we initially trained the model to distinguish between HBM applicable tweets (all HBM constructs combined) and nonapplicable tweets. We further trained the model to classify the top 2 prevalent categories of HBM constructs within the HBM applicable tweets. The speech acts model was trained to classify the top 5 prevalent categories of speech acts. We applied these models to classify HBM constructs and speech act categories from the CMU-MisCov19 data set (n=3702).

#### Social Network Analysis

The CMU-MisCov19 data set was further analyzed using 2-mode network analysis by creating affiliation networks composed of 2 modes (the first mode represented the tweets and the second mode represented the various speech act categories with which the tweets were affiliated). We constructed visual representations of HBM construct-based affiliation networks between tweets and speech acts. We compared the structures and topologies across different networks using various social network metrics such as degree, density, diameter, and average path length. For affiliation networks, degree centrality suggests that an actor (in our case, tweet) is popular because of its membership to certain events (in our case, speech acts), while an event (speech act) is popular based on the size of actors that are part of it [38]. The density of an affiliation network is defined as the number of edges divided by the number of pairs of nodes where only edges between vertex sets are possible [39,40]. The diameter of an affiliation network is the length of the longest path between any pair of actors/events [39,40]. Average path length is defined as the average shortest path between the 2 nodes [39,40]. An open-source network visualization tool, UCINET [41], was used for creating and analyzing these networks.

### Intervention Planning

As part of the IM framework described earlier, our intervention plan included multicomponent strategies, such as use of phone navigation, community health workers, and social marketing, including use of social media. We ensured that the social media intervention is informed by existing empirical evidence, behavioral theories, and new evidence from the needs assessment and social listening.

### Social Media Intervention

To leverage social media to improve the reach of our COVID-19 health promotion materials, we hired a Houston-based creative agency to brand Take Care, Texas (TCT) social media channels tailored to our 3 project regions. The design agency met with project staff experienced in community health promotion to gain insights into population demographics and regional differences, learn about prevalent COVID-19 attitudes, and identify potential structural and psychosocial barriers to accessing COVID-19 testing. Intervention development efforts to counter misinformation necessarily accounted for disparities in COVID-19 morbidity and mortality rates associated with social determinants unique to our 3 project regions. For instance, efforts were adapted in Northeast Texas to account for low population density and in Cameron County to address a largely Spanish-speaking population. Throughout intervention development, community input, including local listening sessions and community scientists’ feedback, helped identify social determinants influencing testing and vaccination behaviors as the situation changed over time. Community scientists also provided feedback on the content and quality of our intervention materials.

In June 2021, our social media team launched 3 social media accounts (ie, Facebook, Instagram, and Twitter) for each region, featuring original content with region-specific design elements and branding (eg, photos that reflect the target population), informational posts without TCT branding, and reposted materials from credible resources. Reposted materials were shared from other health agencies or COVID-19 education efforts including but not limited to the Centers for Disease Control and Prevention, Public Health Media Collaborative, and Vaccine Resource Hub. Posts across the 3 regions overlapped and varied based on local events, demographics, and specific barriers or knowledge gaps identified by community health workers conducting door-to-door outreach. Additionally, community health workers conducted in-person COVID-19 education through door-to-door canvassing, community events with local partners, and COVID-19 testing and vaccination events.

Our regional social media accounts were featured on resource and education materials to direct community members to engage with us online, including door hangers, resource flyers, and maps to COVID-19 testing and vaccination sites. QR codes featured on print materials were also linked to regional social media accounts. In regular meetings with the social marketing team, community health workers shared the most frequently reported barriers influencing residents’ COVID-19 preventive behaviors, which were captured in encounter forms during outreach, to better tailor regional print and online materials. For example, multiple door hanger designs with alternative messaging to promote COVID-19 vaccines were developed and distributed, and included a project phone number through which residents could ask community health workers questions about COVID-19–related information or resources. Once community health workers identified the most effective door hanger for a neighborhood, based on the number of calls received, that messaging was prioritized in the next round of door hanger distribution in that community.

Custom content for all regional accounts included advertisements of local events, residents’ testimonials, and posts that featured each region’s team of community health workers as well as local organizations we partnered with to align and engage with a broader audience. Tailored content on these platforms varied between regions by using images that best reflected each region’s priority population, hashtags that applied to the region, and languages spoken in the region. For example, Spanish was the primary language used on all Cameron County social media accounts and most testimonials from that region included Spanish speakers at testing and vaccination locations in the community.

## Results

### Community Needs Assessment

Our qualitative data collection efforts included 11 interviews with participants, a majority of whom identified as female (8/11, 72%). Themes that emerged from these interviews covered a range of topics, including barriers and facilitators to COVID-19 preventive behaviors (eg, testing, vaccines, and masking), trust in clinical and scientific institutions, the role of technological platforms, the burden of misinformation on general and marginalized populations, and the optimal strategies for risk communication and promoting COVID-19–related health information. All participants mentioned exposure to misleading health information on social media even when they were not actively using it. Distrust in social media as a source of health information was exhibited through descriptions of “algorithmic bias,” “persuasive intent,” and “financial motives,” which in turn created “polarization” and “echo chambers.” These social phenomena were also linked to cognitive heuristics applied by individuals to (1) ensure credibility of information sources through the “reputation” heuristic and (2) limit misinformation exposure through the “self-confirmation” heuristic, and they are aligned with existing literature [42].

Findings from the interviews revealed emotional consequences of misinformation exposure. For example, 82% (9/12) of participants mentioned being “baffled,” “upset,” and “angry” by misinformation, while the rest mentioned “being indifferent” and “losing hope.” Participants linked high levels of perceived confidence in misinformation detection and low levels of perceived vulnerability to academic training, their ability to apply cognitive heuristics, such as reputation (to assess source credibility), and their life experiences associated with age (Table 1). Participants often linked vulnerability to the emotional distress caused by challenging circumstances such as the diagnosis of a chronic or terminal condition such as cancer. Participants emphasized the need to be self-reliant, rather than dependent on platform-based misinformation flagging or third-party fact checking tools, given issues with the time-sensitive, evolving, and emotional nature of information-seeking patterns.

Data from the listening sessions elicited similar themes, with fear of the vaccine emerging as a barrier to vaccination. Most fears stemmed from misinformation circulated on social media concerning side effects, the content of the vaccine, and the rushed approval of the vaccine. Another common barrier was mistrust in the government and public officials recommending the vaccine. As seen in the examples in Table 1, participants exhibited awareness, helplessness, and confusion stemming from the pervasive misinformation they encountered in online social media, which they attributed to the financial motives and algorithmic shortcomings of these platforms. Participants mentioned that there is a possibility of echo chambers and polarization in the name of targeted advertising and personalization for extended digital engagement, which they experienced firsthand when using social media.

Community scientists also highlighted the unjust burden misinformation puts on vulnerable populations by widening the disparities that already affect health outcomes and quality of life. Community scientist sessions and stakeholder interviews resulted in common themes related to fear, side effects, worsened outcomes, and tracking devices, enabling us to identify information sharing mechanisms to combat misinformation. One suggested tactic was to provide a frequently asked questions (FAQ) post with a QR code attached with expertise and insight from physicians and other health care professionals.

**Table 1:** Comments and feedback from interviews and listening sessions.

Theme	Comments and feedback
Attitudes toward social media misinformation	I'd say it affects—misinformation definitely makes me upset. I guess I get kind of irritated and angry. [Younger White female] I stopped using social media because it is harmful for mental health and wellbeing, a waste of time. [Older South Asian male] ...not to really just become something to reach a consensus because that’s not happening in today’s social media. Nobody gets into social media or into a conversation with the intent of coming to a logical conclusion. [Older South Asian male]
Social media as an echo chamber	But, you know, if people aren't necessarily aware of or accepting of the fact that they are in an echo chamber, then it's much more difficult. [Younger Hispanic male] My Facebook generally does tend to be in an echo chamber. I think it's something that, that I'm aware of. [Older South Asian female]
Individual vulnerability and confidence to detect misinformation	Or people who are not really IT guys. They look at something and they get influenced by this. They think the technology is helping validate what they already know, no it is reading you, been there done that. [Older Asian male] I'm going to say moderately confident. Sometimes, I have to tell myself, if it's too good, if it looks too good to be true, it's just too good to be true. Check more, google more [Younger Hispanic female]
Burden of misinformation on marginalized groups	The burden of misinformation is definitely higher in low-income and minority women who may not have access to care or information resources that are credible. [Older Black female] No one can get accurate information that is sure shot, how can people with no insurance trust anything they see. It is difficult, no information is better than wrong information. I don’t know, never so unsure my life. [Older Asian male]
Misinformation example	There are many myths that you don't know if they are true or lies. … supposedly, there’s a chip that people want to put in to see where you are located. And …, they were supposedly putting a chip on the skin with the vaccine. [Younger Spanish-speaking adult] Vaccines can cause blood clots, one of my close buddies passed away, I am already at higher risk, can’t risk more. [Older Hispanic female]
Lack of trust in science and clinical institutions	Mistrust in the U.S. health system, including the Tuskegee experiments and concerns around population control. [Younger Black male] When you keep changing words, when so much drama in news, difficult to trust. [Older South Asian male]
Role of community leaders	I learn from trusted messengers, especially my faith leader. [Older Black female] Never know whom to trust, I see no leadership, just vacuum top to bottom. [Younger White male]
Design ideas for health communication	This is like highway driving, having warning signs might help. [Older South Asian male] One of my friends just doesn’t want to learn, not sure how we can make her see. Someone she trusts and can’t block like she does in Facebook. Information in the face she can’t ignore. [Younger Hispanic male]

### Online Social Listening

The most common health belief was perceived severity (369/1400, 26.4%), showing that most of the messages reflected individuals’ concerns about the growing severity of the virus (eg, “Coronavirus infections predicted to grow exponentially; first death outside China; outbreak becomes political”). Perceived barriers (239/1400, 17.1%) were also prevalent, reflecting how individuals experienced barriers to performing COVID-19–related prevention behaviors, such as mask-wearing, quarantining, and testing (eg, “I think CNN needs to focus on figuring out how to correct the day-to-day operations and start telling the truth about irrelevance of COVID-19 testing!”). Other health beliefs were expressed in the form of perceived susceptibility (83/1400, 5.9%; eg, “With that healthy asymptomatic person and contract a more severe form of COVID19, then who is responsible for me getting sick?”) and perceived benefits (37/1400, 2.6%; eg, “Can’t break out the champagne yet, but efforts to avoid a surge have been working, in some jurisdictions”). In terms of speech acts, the most prevalent speech act was assertion (445/1400, 31.8%) where individuals expressed their beliefs about the spread of the virus (eg, “If the UK doesn't go on lockdown virtually now we are going to be in the same position as Italy in another week or two”), followed by declaratives (373/1400, 26.6%) about objective information related to COVID-19 (eg, “The CDC is now performing entry health screening on all passengers with direct and connecting flights from Wuhan, China”). Directives (300/1400, 21.4%) in the form of advice about what precautions one should take were also common (eg, “We need to take things seriously, … and follow the advice of the medical professionals”). Tweets also posed questions (204/1400, 14.6%) regarding concerns about COVID-19 using Twitter (eg, “Can you contract COVID-19 from a mosquito?”).

Table 2 shows the F1 scores of deep learning models on the various classification tasks. For HBM construct classification, the ensemble model achieved the highest F1 score for every category and for the overall macro average (0.81) of the model in comparison to the BERTweet model or the BERTweet-Covid19 model. The BERTweet model achieved the highest F1 score for every category of speech acts and for the overall macro average (0.80) of the model in comparison to the BERTweet-Covid19 model or the ensemble model.

**Figure 2:**
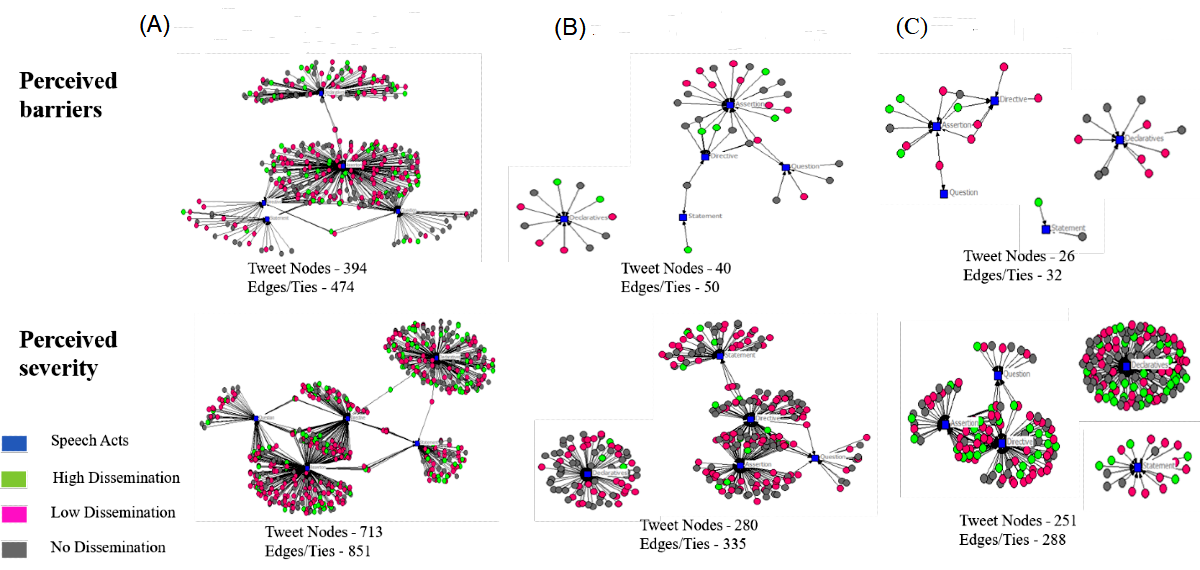
Two-mode affiliation networks for (A) misinformation correction tweets, (B) false information tweets, and (C) true information tweets within the 2 Health Belief Model constructs.

For illustration purposes, we compared the 2-mode affiliation networks of tweets and speech act expressions for stance taking misinformation correction tweets, false information tweets (ie, tweets annotated for the labels fake cure, fake treatment, false public health response, and false fact or prevention), and true information tweets (ie, tweets annotated for the labels true public health response and true prevention) within the two HBM constructs (ie, perceived barriers and perceived severity) in Figure 2. In these networks, the tweets’ nodes were colored based on their dissemination levels, with blue nodes representing the different speech act categories (Figure 2).

Table 3 shows the network metrics calculated for the various 2-mode networks. Within the perceived barriers health belief, the stance taking misinformation corrective tweets network had the highest average path length, indicating that the efficiency of information transfer expressed using a certain category of speech act was low as compared to the other 2 networks. Given the density and path length of the false information network, the circulation of false information pertaining to health-related barriers about COVID-19 expressed via speech act categories was much faster, whereas the corrective information about health-related barriers regarding COVID-19 took longer to travel within the network as per our data set. In the stance taking misinformation corrective tweets network, the high dissemination tweets had a higher prevalence of assertion and declarative speech acts within the perceived barriers health belief, whereas the high dissemination tweets had a higher prevalence of declarative speech acts within the perceived severity health belief. Thus, misinformation correction strategies should focus on integrating declaratives (eg, provide objective information) within their messages to have a greater impact on the online community of users who are exposed to misinformation.

Within the perceived barriers HBM construct networks, the most commonly used speech acts within the stance taking misinformation correction tweets network were assertion (degree=0.635) and declaratives (degree=0.246). The most commonly used speech acts within the false information tweets network were assertion (degree=0.575) and declaratives (degree=0.275). The most commonly used speech acts within the noncorrective tweets network were assertion (degree=0.423) and declaratives (degree=0.423). Within the perceived severity HBM construct networks, the most commonly used speech acts within the stance taking misinformation correction tweets network were assertion (degree=0.446) and declaratives (degree=0.339). The most commonly used speech acts within the false information tweets network were assertion (degree=0.339) and declaratives (degree=0.279). The most commonly used speech acts within the noncorrective tweets network were declaratives (degree=0.510) and assertion (degree=0.203). Even though the degrees for speech act assertion and declaratives were higher in all 3 networks, the higher values within the stance taking corrective network indicated that tweets were more prominently expressing those speech acts compared to the other 2 networks. ANOVA revealed that there was a statistically significant difference in the degree centrality of tweet nodes between the 3 dissemination levels (*P*=.006) for perceived severity HBM construct-based networks.

**Table 2:** Evaluation (F1 scores) of deep learning models for the classification of Health Belief Model constructs and speech acts.

Variable				BERTweet		BERTweet-Covid19		Ensemble (BERTweet+BERTweet-Covid19)
**Health Belief Model constructs**								
	**Per class performance**							
		Perceived barriers	0.64		0.64		0.77	
		Perceived severity	0.76		0.80		0.83	
	**Overall model performance**							
		Macro average	0.71		0.74		0.81	
**Speech acts**								
	**Per class performance**							
		Assertion	0.80		0.78		0.82	
		Declaratives	0.92		0.83		0.89	
		Directive	0.80		0.73		0.67	
		Question	0.83		0.88		0.83	
		Statement	0.67		0.59		0.69	
	**Overall model performance**							
		Macro average	0.80		0.76		0.78	

**Table 3:** Metrics for various Health Belief Model construct-based 2-mode affiliation networks.

Construct and tweet network type			Average path length	Diameter		Density
**Perceived barriers**						
	Stance taking misinformation corrective	3.158		6	0.241	
	False information	2.635		6	0.250	
	True information	2.095		5	0.246	
**Perceived severity**						
	Stance taking misinformation corrective	3.769		6	0.239	
	False information	3.185		6	0.239	
	True information	2.216		4	0.229	

### Outputs From IM Framework Application

We identified several behavioral science constructs and determinants associated with individuals’ intentions to test for COVID-19, intentions to receive COVID-19 vaccinations, attitudes toward testing for COVID-19, and attitudes toward receiving COVID-19 vaccinations, including constructs from the HBM and the Theory of Planned Behavior [32]. Table 4 provides a list of the key constructs and determinants that we used to develop TCT social media content.

**Table 4:** Behavioral theory constructs and example social media content to promote COVID-19 testing and vaccination behaviors.

Model, COVID-19–related behavior, and construct				Definition		Example messaging		Implementation (social media post examples)
**Health Belief Model [32]**								
	**COVID-19 testing and vaccination**							
		Perceived susceptibility	Belief in the likelihood of getting an illness or disease.		“The Omicron variant is spreading in our community. Anyone can get it.”* *		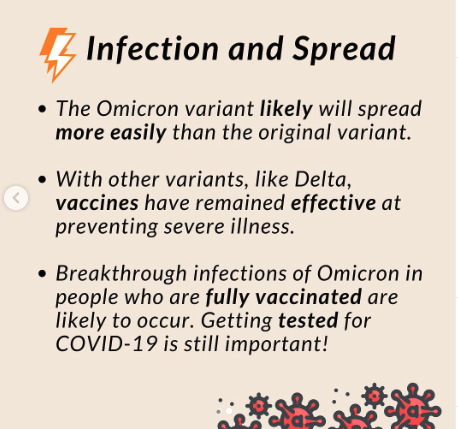	
	**COVID-19 vaccination**							
		Perceived severity	Belief in the severity of an illness or disease.		“Unvaccinated COVID-19 patients have a greater risk of hospitalization and death.”		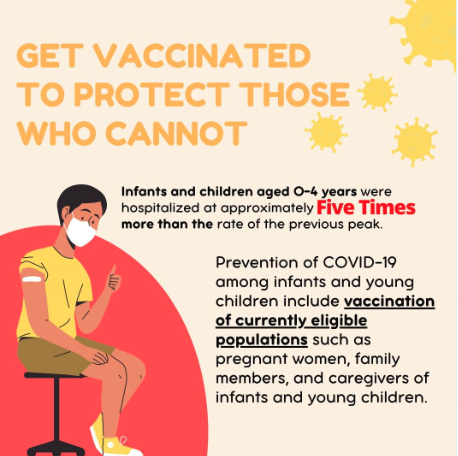	
		Perceived barriers	Belief in the obstacles that impede performing the behavior of interest.		“Getting the vaccine is easy. Many drug stores have walk-in appointments.”		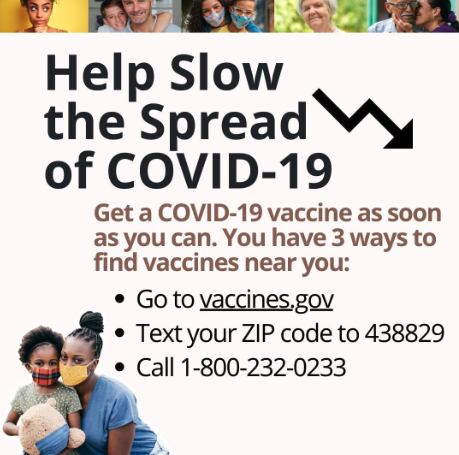	
		Perceived benefits	Belief in the effectiveness of the behavior of interest.		“COVID-19 vaccines are free, safe, and effective.”		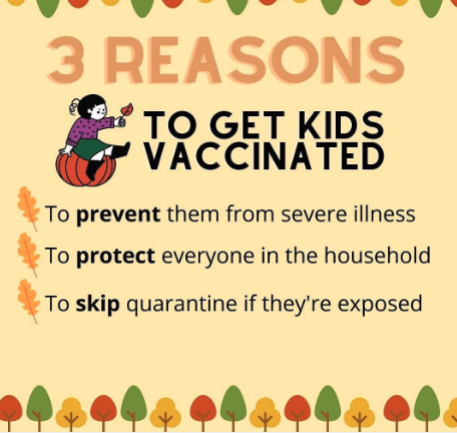	
	**COVID-19 testing**							
		Self-efficacy	Confidence in the ability to perform the behavior of interest.		“It’s easy to place your order for free at-home COVID-19 tests at covid.gov. I did it in 5 minutes.”		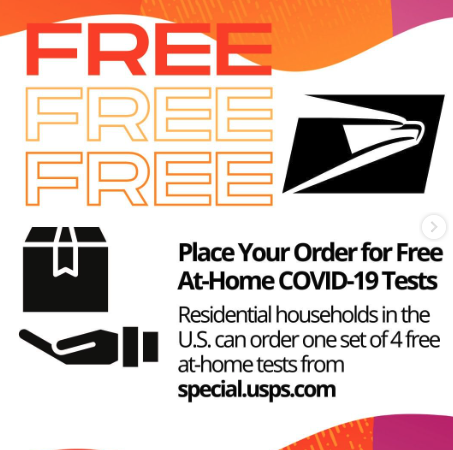	
**Theory of Planned Behavior [32]**								
	**COVID-19 testing**							
		Attitudes	Favorable or unfavorable evaluation of the behavior of interest.		“Regular testing can help give your family peace of mind.”		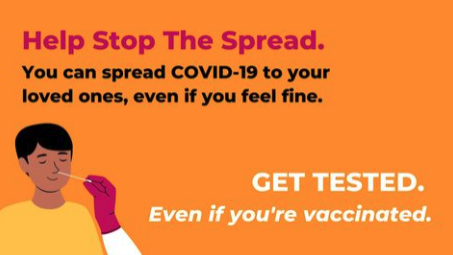	
	**COVID-19 vaccination**							
		Subjective norm	Belief about whether important others (parents, partners, or doctors) approve or disapprove of the behavior of interest.		“I vaccinate because I want to keep my children safe. It’s the same reason that they wear helmets and seatbelts. #WhyIVaccinate” “I got vaccinated because everyone in my family thought it was an important thing to do. #WhyIVaccinate”		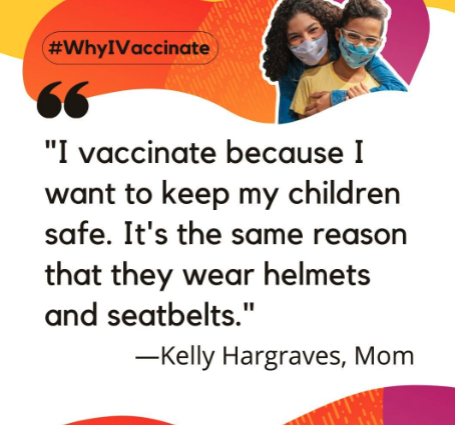	

### Social Media Interventions and Dissemination

We describe here the cumulative number of posts as well as the engagement metrics for each TCT account in each project region from June 2021 through March 2022, with marked regional differences in engagement by platform. On Facebook, Northeast Texas had the greatest number of posts (n=257), but Cameron County had the largest numbers of fans who liked and followed the account (n=246 and 250, respectively). Cameron County also had the greatest numbers of shared posts and video views (n=38 and 81, respectively), while the Harris County account had the greatest number of reactions (n=116). On Twitter, Northeast Texas had the most tweets (n=332), but Harris County had the greatest number of followers (n=19). While Northeast Texas had the greatest number of replies (n=57), Harris County had the greatest numbers of likes and retweets (n=105 and 95, respectively). On Instagram, Northeast Texas had the most posts (n=233). The Northeast Texas account also had the most community engagement with 440 likes and 2 comments, although the Harris County account had the most followers (n=111). Overall community engagement was determined by the numbers of likes and comments that each post or tweet received. Examples of posts/tweets with high amounts of community engagement for all of the accounts across the 3 regions include informational posts on the virus and its variants (Delta and Omicron), testing and vaccination/booster guidelines (when to test, which vaccine/booster to get, etc), masking guidelines, and community member testimonials.

**Figure 3:**
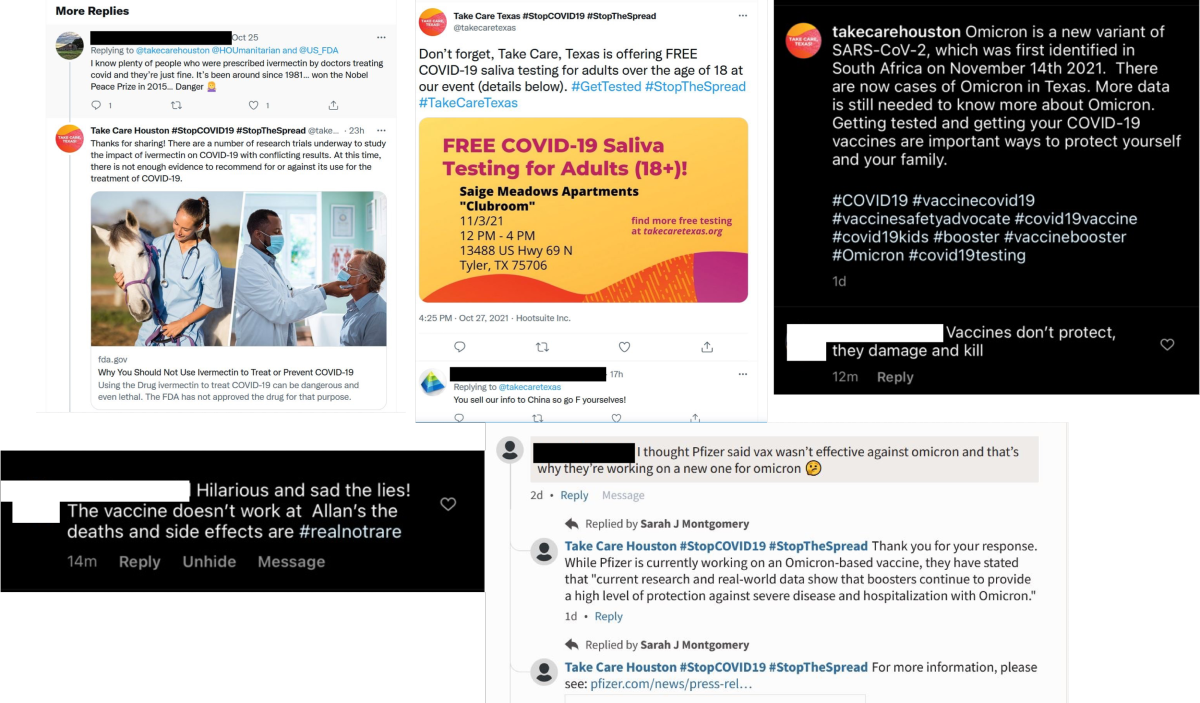
Sample bullying and misinformation posts on Take Care, Texas social media channels.

However, not all community engagement was positive (Figure 3). Some posts, especially those responding to myths, received comments containing misinformation. Examples of such posts include a post debunking the use of ivermectin as a treatment for COVID-19, a post about the Omicron variant, a post about vaccination, a post advertising a testing event in Northeast Texas, and a post debunking the myth that COVID-19 vaccines change a person’s DNA. One of the following 3 actions was taken in response to these comments: (1) We attempted to educate the user who made the comment by providing information from reputable sources; this action was taken for comments on a post debunking a myth about the COVID-19 vaccine and a post debunking the use of ivermectin as a treatment for COVID-19; (2) We ignored the user; this action was taken for comments responding to a post about vaccination and a post about the Omicron variant; and (3) We deleted the comment and blocked the user from replying to other posts; this action was taken for comments responding to a post about a testing event in Northeast Texas, as the comment included offensive language.

The analysis of social media posts provided us with insights into the latent needs of the users via the expression of various categories of speech acts based on their health beliefs toward COVID-19 misinformation. Such insights can be translated to design the architecture of just-in-time adaptive interventions, such as chatbots, thus ensuring such virtual interventions are theory-informed and data-driven for efficiently combating COVID-19 misinformation.

## Discussion

### Principal Findings

Table 5 presents an overview of the key findings and contributions from the study. Our results from community needs assessment indicated that participants from minority communities mentioned routine social media use and misinformation exposure, and emphasized the increased burden of misinformation among vulnerable populations. These findings enabled us to reposition online social media as a crucial data resource to understand COVID-19 misinformation dynamics not just among the general population, but also among minority groups. This finding is supportive of existing research that indicates no significant racial/ethnic disparities in social media use [43]. The analysis of needs assessment data and social media data sets can provide us with an opportunity to extend the application of human communication models to design informative and credible public health messages for risk communication with the potential to reduce public health burden and inform policy regulations. Such analytical insights can be translated for developing consumer education tools to improve the health literacy of community members such that there is a higher likelihood of individuals recognizing and resisting misinformation. So far, few misinformation mitigation interventions have effectively packaged theory and data insights simultaneously [44-48]. These data-driven theory-guided approaches are important during life-threatening public health events such as the COVID-19 pandemic.

Our results from social media listening and context-aware network analysis portray distinct network topologies and properties, and their dependencies on the semantics and syntactics of human communication [49]. The communication content, intent, structure, and framing together define the persuasive aspects of a social media information facet [50]. Using these tools in the engineering of risk communication can enable us to channel information efficiently, allowing us to target individuals and populations through personal and social contexts using social media.

As evident in our dissemination work, our efforts to promote COVID-19 testing and vaccination reach have been underwhelming. As part of our future efforts, we will apply our online social listening techniques to TCT data to examine content-based and network-driven facilitators and barriers to community engagement. Recruitment of advertising agencies and micro- and macro-influencers has become the new norm for implementation science in health promotion. The budgetary overhead of advertising costs to achieve adequate reach in social media has been concerning, especially for rural public health programs with limited resources, which can be remediated to some extent through the provision of advertising credits (as is done with cloud service credits for National Institutes of Health–funded projects) and reconfiguration of ranking algorithms that promote public health posts from verified scientific sources and nonprofit organizations, providing clear and direct-to-consumer digital engagement pathways for science in social media [51,52]. It is essential to facilitate community investments and educational offerings for social media optimization of outreach and prevention activities with a specialized focus on health marketing techniques, media literacy, and peer modeling. Specifically, prioritizing social media advocacy roles in public health and nonprofit organizations is imperative. One way to support this important function is through curriculum offerings from social media corporations with training on platform-specific optimization processes and financial monetization pathways for sponsored and nonsponsored posts by influencers in social media.

Millions of posts and accounts have been deleted and suspended across multiple social media platforms to combat misinformation spread on social media [53]. To support open science and agile science endeavors in this pertinent topic, we suggest online social media platforms release deidentified batches of social media posts so transdisciplinary theories, data analysis techniques, and innovative social marketing and data science methodologies can be developed and applied to understand the sociotechnical dimensions of misinformation spread [54]. Linking with location-sensing attributes can map misinformation spread to identify communities with the most dissemination. Such modeling efforts can enable us to optimize the positive supportive facets of online social media while examining its unintended consequences of polarization and social chambers [55]. Constant dialogue with community members and scanning of social media are essential to stay relevant and aware of misinformation [56]. Our work has done this, although this paper reports the data from a single time period. The resources and time to acheive sustainability of such efforts are extensive, whereas intentional and unintentional diffusion of misinformation appear to be much less demanding.

Our theoretical contributions are as follows. We built a foundational step to characterize the dynamics of health beliefs and intentions in online social media platforms to develop resilient information dissemination approaches that are resistant to misinformation spread [49,50]. We extended and applied behavioral constructs, which were developed in the era of face-to-face communication, to digital interactions on social media [57]. Our practical contributions include the development of end-to-end full stack social marketing materials using the IM framework, which could be repurposed for other health domains and community settings. Further, our findings from social media analysis can help us understand the mechanisms of social influence and content-specific patterns, which will help us engineer better content development and diffusion strategies.

**Table 5:** Key findings and contributions from this study.

Methodology	Key findings
Community needs assessment	Several themes regarding COVID-19 misinformation were highlighted in this study, including barriers and facilitators to preventive behaviors, the role of technology, etc. We also identified that social media is a primary resource of information and source of misinformation among minority communities.
Online social listening	This study provided population-level insights and patterns underlying behavioral constructs, communication attributes, and online social ties. Specific network structures and content forms were found to be efficient vehicles for misinformation resistance and true information dissemination. Significant differences were found between different expressions and content areas using online social listening, which can guide the development of impactful risk communication messages and expert conversational agents harnessing naturalistic conversational attributes as expressed in online social media.
Social marketing	Rigorous community needs assessment and theory integration allowed us to curate and develop a portfolio of social marketing materials, including social media postings. Despite evidence-based communication methods, community engagement and traction in online social platforms were limited.

### Limitations

Our work is not without limitations. The interviews and listening sessions were conducted after May 2021, while the Twitter data collection covered the time period from January 2020 to January 2021. However, our analysis of Twitter data allowed us to capture key mechanisms underlying peer interactions when discussing COVID-19–related health behaviors. Results from our analysis have both mechanistic and topical findings (eg, how do we structure an interventional message vs what is the trending misinformation topic in a given time frame?). While we used our findings from social listening to inform interview questions and focus group guides, agile integration would further enhance the impact of our methods. Further, we included only the top 5 most prevalent speech act classes to train our deep learning model for the classification of the corrective misinformation Twitter data set, which led to the omission of the remaining speech act classes. In addition, while we only illustrated HBM constructs in the paper for online social listening, we have been using an integrated model with multiple behavior theories in our ongoing work. Not all the tweets were retrieved when the data sets were hydrated because of the retrospective organizational review policies, such as deleted tweets or user account suspensions. Some inherent challenges in using Twitter as the data source include restrictions on the number of requests/calls that can be made to the Twitter API, which leads to increased costs in obtaining the entirety of the data, fictitious or fake accounts, and privacy and ethical issues. Our sample size in participant interviews was limited, although additional data were collected in the form of listening sessions and focus groups. The developed interventions, which include social marketing materials, preliminary chatbot architecture, and social media content, still need to be evaluated for their effects toward combating misinformation.

### Conclusions

We described a series of community-based studies and a large-scale observational study to examine and intervene regarding COVID-19 misinformation in our society and its vulnerable populations. While the internet and social media have democratized information, providing tools to marginalized groups to combat misinformation (ie, digital wildfires) is crucial [58]. Our study highlights the potential of online social listening to develop impactful risk communication strategies to combat misinformation spread and seeding on social media platforms. The feasibility and integration of data-intensive and grassroot-focused social engineering can provide a wealth of tools to ensure our communities are aware of and empowered with the skills of social media literacy to stay alert of cognitive blind spots (ie, heuristics) inherent in human reasoning with information environments [59,60]. For us to achieve the scalability and sustainability of social media operations in public health, it is important for industry corporations to provide the government and nonprofit organizations with technical resources and financial incentives along with algorithmic retraining to upvote evidence-based content for public wellness.

## Abbreviations

API: application programming interface
HBM: Health Belief Model
IM: Intervention Mapping
TCT: Take Care, Texas
